# Effect of Source/Drain Electrodes on the Electrical Properties of Silicon–Tin Oxide Thin-Film Transistors

**DOI:** 10.3390/nano8050293

**Published:** 2018-05-02

**Authors:** Xianzhe Liu, Honglong Ning, Weifeng Chen, Zhiqiang Fang, Rihui Yao, Xiaofeng Wang, Yuxi Deng, Weijian Yuan, Weijing Wu, Junbiao Peng

**Affiliations:** 1 State Key Laboratory of Luminescent Materials and Devices, South China University of Technology, Guangzhou 510640, China; msliuxianzhe@mail.scut.edu.cn (X.L.); ninghl@scut.edu.cn (H.N.); chenweifengchn@foxmail.com (W.C.); ilttplws@163.com (Y.D.); g18826075867@163.com (W.Y.); psjbpeng@scut.edu.cn (J.P.); 2 State Key Laboratory of Pulp and Paper Engineering, South China University of Technology, Guangzhou 510640, China; fangzq1230@126.com; 3 Institute of Semiconductors, Chinese Academy of Science, Beijing 100083, China; wangxiaofeng@semi.ac.cn

**Keywords:** source/drain electrodes, thin film transistors, Si-doped SnO_2_, density of states

## Abstract

Ultra-high definition displays have become a trend for the current flat plane displays. In this study, the contact properties of amorphous silicon–tin oxide thin-film transistors (a-STO TFTs) employed with source/drain (S/D) electrodes were analyzed. Ohmic contact with a good device performance was achieved when a-STO was matched with indium-tin-oxide (ITO) or Mo electrodes. The acceptor-like densities of trap states (DOS) of a-STO TFTs were further investigated by using low-frequency capacitance–voltage (C–V) characteristics to understand the impact of the electrode on the device performance. The reason of the distinct electrical performances of the devices with ITO and Mo contacts was attributed to different DOS caused by the generation of local defect states near the electrodes, which distorted the electric field distribution and formed an electrical potential barrier hindering the flow of electrons. It is of significant importance for circuit designers to design reliable integrated circuits with SnO_2_-based devices applied in flat panel displays.

## 1. Introduction

Amorphous oxide semiconductor thin-film transistors (AOS-TFTs) as candidates for silicon-based TFTs have come into the spotlight of flat panel display (FPD) because of their low processing temperature, high transparency, high carrier mobility, and good uniformity [1,2,3]. In 2012, the implementation of mass production of FPD with the amorphous In–Ga–Zn–O (a-IGZO) TFT backplane was a milestone for the development of AOS-TFTs [4]. The requirements of high-resolution, large-size, and narrow border displays are pursued with the advances in display technology. For achieving these requirements, miniaturized TFT with a suitable structure, a high mobility, and a low contact resistivity should be fabricated, which could eliminate image distortion and shading effects during operation. In the conventional AOS-TFTs structure, an etch stopper layer (ESL) should be inserted between the semiconductor and the source/drain (S/D) electrodes to protect the channel layer from damage during the etching of the S/D electrodes. The miniaturized device can be hardly fabricated by this ESL configuration. Compared to the ESL configuration, a TFT with a back-channel-etched (BCE) structure is preferable to achieve the miniaturized fabrication of the AOS device. However, most of the AOS are sensitive to weak acid, which would limit their applications to large-size TFT backplanes. Fortunately, acid-resistant SnO_2_ has been suggested as a potential candidate, and the device can obtain a high mobility because of the electronic configuration of the tin ions and indium ions. In previous work, the feasibility of silicon-doped tin oxide (a-STO) TFTs with BCE structure has been demonstrated [5]. However, the contact characteristics of a-STO TFTs are still unknown. Therefore, it is very necessary to investigate the contact properties of a-STO with different electrodes and facilitate its practical industrial application.

Until now, some interesting phenomena related to the contact properties of AOS-TFTs, such as self-formed metal-oxide contact interlayer [6,7] and metal atom diffusion [8] or migration [9], have been reported in detail. However, there is no in-depth understanding about how S/D electrodes affect the device performance. Therefore, it is significant to explore whether the S/D electrode could affect the density of states (DOS) of a device. In AOS devices, the subgap DOS is an important parameter which plays a major part in controlling the mobility, operation voltage, and subthreshold swing of TFTs [10]. Up to now, many extraction methods of DOS based on AOS-TFTs have been developed in numerous studies. For example, Kimura et al. directly extracted the DOS on the basis of the numerical iterative solution of Poisson’s equation by fitting the measured and calculated capacitance–voltage (C–V) characteristics of a-IGZO TFTs at extremely low frequencies, in which the free carrier density had not been taken into account [11]. Park et al. have proposed the extraction method of acceptor-like DOS of a-IGZO TFTs by combining subgap optical charge pumping and C–V characteristics and verified the measured characteristics compared with Technology Computer Aided Design (TCAD) simulation results incorporating the extracted DOS [12]. Bae et al. have proposed a physics-based generation–recombination current spectroscopy of n-channel a-IGZO TFTs for extracting the deep donor-like DOS, which is verified by comparing the calculated generation–recombination current with the measured one [13]. Chen et al. calculated the DOS of a-IGZO TFTs by a straightforward method based on temperature-dependent field-effect measurements in consideration of the Meyer–Neldel rule [14]. However, the instability of TFTs caused by illumination or thermal effect was ignored during the extraction of the density of states. Consulting the abovementioned methods, a viable and effective method was adopted to extract the DOS of a-STO TFTs.

In this paper, the contact characteristics of a-STO TFTs with different S/D electrodes, including ITO and Mo, were studied. For the first time, the DOS of acceptor-like states in a-STO TFTs was extracted by using low-frequency C–V characteristics.

## 2. Materials and Methods

TFTs with BCE structure were fabricated on a glass substrate. A 300-nm-thick Al–Nd alloy (3 wt % of Nd) as gate metal was deposited by direct current (DC) magnetron sputtering, and its patterns were defined by photolithography. Then, the Al–Nd alloy film was anodized to form a 200 nm dielectric layer of AlO_x_–Nd on the surface in an electrolyte consisting of 3.68 wt % ammonium tartrate solution and ethylene glycol. Afterwards, a 5-nm-thick a-STO film was deposited by radio frequency (RF) magnetron sputtering (SNTEK) with a SiSnO (SiO_2_/SnO_2_ = 5:95 wt %) ceramic target on the dielectric anodized alumina. For the S/D electrodes, ITO (150 nm) and Mo (200 nm) were deposited by DC magnetron sputtering at room temperature, and the channel length/width (100/30 μm) was patterned by a wet etching process. All the devices were subjected to thermal annealing at 350 °C on a hotplate for 0.5 h in ambient air. At last, a passivation layer of SiO_2_ (300 nm) was deposited by using a plasma-enhanced chemical vapor deposition (PECVD). In order to obtain high performance of the a-STO TFTs, the devices were annealed at 450 °C for 0.5 h in a protective argon ambient.

The thickness of a-STO films deposited on the glass substrate were measured by X-ray reflectivity (XRR, Empyrean, PANalytical, Almelo, The Netherlands) (See in Figure S1 and Table S1). The electrical characteristics of TFTs were measured using a semiconductor parameter analyzer (Agilent, 4155C, Santa Clara, CA, USA) in the dark in ambient air. The positive gate bias stress (PBS) stability of the devices were measured under the following conditions: *V*_GS_ = *V*_DS_ = 30 V. The capacitance versus voltage (C–V) curve of a-STO TFTs was measured by a KEYSIGHT E4990A Impedance Analyzer (Keysight Technologies Inc., Santa Rosa, CA, USA) with a frequency of 10k Hz. The device simulation was performed by the 2D device simulator ATLAS (Silvaco Inc., Santa Clara, CA, USA). The properties of the channel region of the device and Mo/a-STO interface were analyzed by transmission electron microscope (TEM, Bruker, Adlershof, Berlin, Germany) (See in Figures S2 and S3).

## 3. Results and Discussion

**Figure 1:**
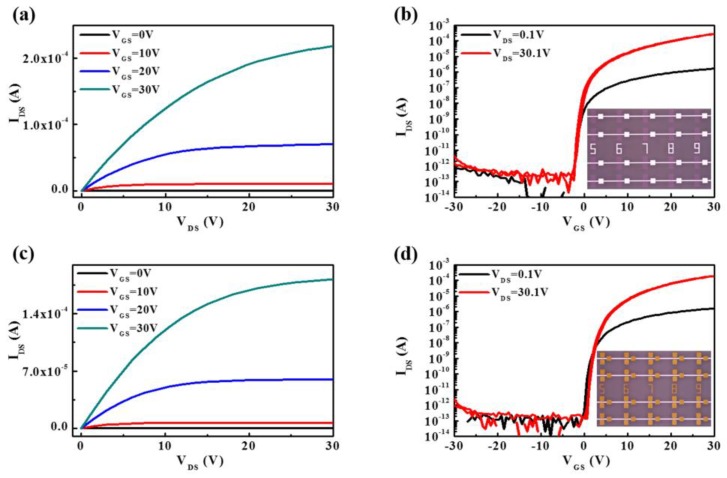
The representative output characteristic curves of amorphous silicon–tin oxide thin-film transistors (a-STO TFTs) with different source/drain (S/D) electrodes: (**a**) ITO and (**c**) Mo. The corresponding transfer characteristic curves with (**b**) ITO and (**d**) Mo.

Figure 1 shows the representative output and transfer characteristic curves of a-STO TFTs with ITO and Mo as S/D electrodes. TFTs with ITO and Mo contact showed excellent drain current characteristics without current crowding, which indicated that Ohmic contact could be formed at the electrode/a-STO interface. A good transfer curve of ITO or Mo without hysteresis could be obtained. Thus, the field effect mobility (*μ*_FE_) in the linear region and the saturated mobility (*μ*_sat_) in the saturation region of the device were extracted by using the following Equations: [2]

(1) $$I_{\mathrm{DS}}=\frac{W}{L}\mu_{\mathrm{FE}}C_{i}(V_{\mathrm{GS}}-V_{\mathrm{th}}-\frac{1}{2}V_{\mathrm{DS}})V_{\mathrm{DS}}$$

(2) $$I_{\mathrm{DS}}=\frac{W\mu_{\mathrm{sat}}C_{i}}{2L}{\left(V_{\mathrm{GS}}-V_{\mathrm{th}}\right)}^{2}$$

where *V*_DS_ is the drain voltage, *V*_GS_ is the gate voltage, *V*_th_ is the threshold voltage, *W*/*L* is the channel width/length, and *C*_i_ is the gate capacitance per unit area of the insulator layer. The subthreshold swing (*SS*) could be calculated using the following Equation: [3]

(3) $$SS={\left(\frac{d\log(I_{D})}{dV_{G}}\right)}^{-1}$$

To ensure the actual level of the electrical properties of the devices with different electrodes contact, four devices were randomly chosen and measured for each S/D electrode material. The electrical parameters of the devices, including field-effect mobility (*μ*_FE_), saturation mobility (*μ*_sat_), turn-on voltage (*V*_on_), on/off current ratio (*I*_on_/*I*_off_), and subthreshold swing (SS) are list in Table 1. The *μ*_FE_ of the a-STO TFTs were 5.6 ± 0.9 cm^2^/V s (ITO) and 5.4 ± 0.2 cm^2^/V s (Mo). Theoretically, Ohmic contact would be formed in the two devices on the basis of the energy band structure of a-STO film in contact with ITO and Mo (see in Figures S4 and S5). In consideration of all devices subjected to the same thermal annealing process, the slight difference of electrical characteristics of the two devices was originated in the contact quality at the S/D electrode–a-STO interface.

**Figure 2:**
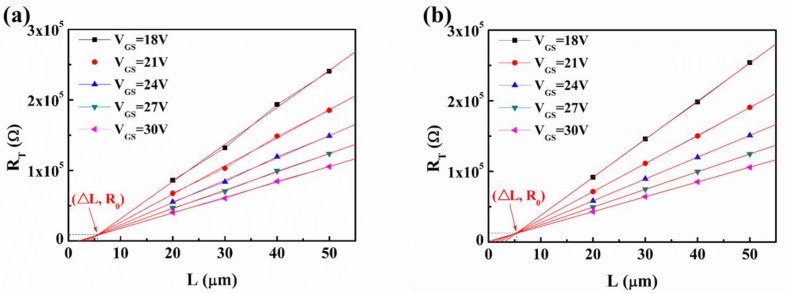
Plot of the total resistance (*R*_T_) versus the channel length (*L*) for a-STO TFTs with different S/D electrodes: (**a**) ITO and (**b**) Mo. The fixed channel width (*W*) is 100 µm, and the various channel lengths (*L*) are 20, 30, 40, and 50 µm.

**Figure 3:**
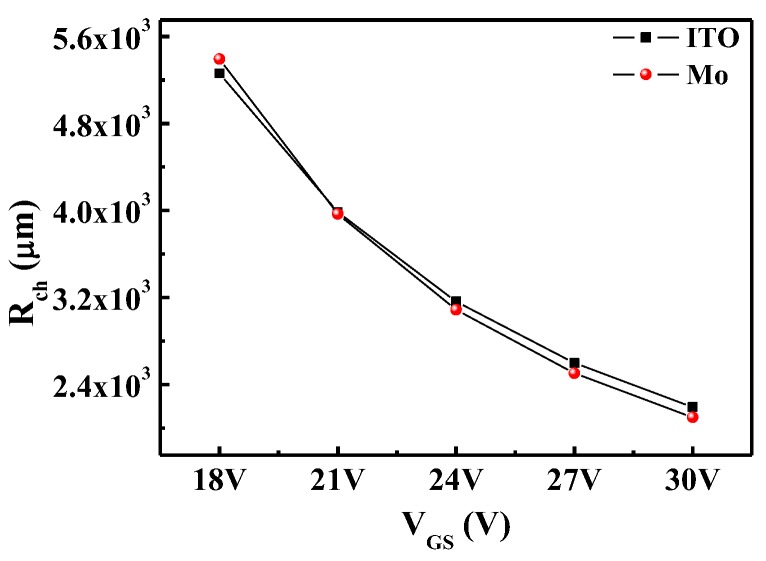
Plot of the channel resistance per unit channel length (*R*_ch_) of a-STO TFT with ITO and Mo contact as a function of the gate voltage (*V*_GS_).

To analyze the reasons for the variation of the electrical characteristics of the a-STO TFTs, the contact properties between a-STO and the electrode were examined using the transmission line method (TLM) [15]. The total resistance (*R*_T_) as a function of channel length (*L* = 20, 30, 40, and 50 μm) for different *V*_GS_ values were plotted, and then the experimental values were fit linearly for each *V*_GS_ value, as illustrated in Figure 2. The *R*_ch_ and *R*_SD_ were extracted by the following Equation (4) in the linear regime:

(4) $$R_{T}=R_{\mathrm{ch}}L+R_{\mathrm{SD}}=\frac{L+2\Delta L}{WC_{i}(V_{\mathrm{GS}}-V_{T})\mu_{\mathrm{FE}-i}}+R_{\mathrm{SD}}$$

where *R*_T_ is the total resistance, *R*_ch_ is the channel resistance per unit channel length, *R*_SD_ is the series resistance at the S/D contacts, *C*_i_ is the capacitance per unit area, *μ*_FE-i_ is the intrinsic field-effect mobility, *L* is the physical channel length, and ∆*L* is the difference between the effective channel length and the physical channel length (*L*) at the S/D contact. Interestingly, an intersection point, providing the resistance (*R*_0_ = 0.5*R*_SD_) value and ∆L, was found in a-STO TFTs with ITO and Mo electrodes, respectively. The *R*_0_/∆*L* was 4193.7 Ω/2.81 μm (ITO) and 5871.1 Ω/2.54 μm (Mo). On the basis of Equation (4), *R*_ch_ is obtained from the slope. By plotting the *R*_ch_ of a-STO TFTs with ITO and Mo electrodes as a function of *V*_GS_, two curves were nearly identical, as shown in Figure 3. This indicated that the consistency of the channel layer’s property could be guaranteed in the two devices. Herein, the different electrical performances of a-STO TFTs were caused by the contact resistance.

**Figure 4:**
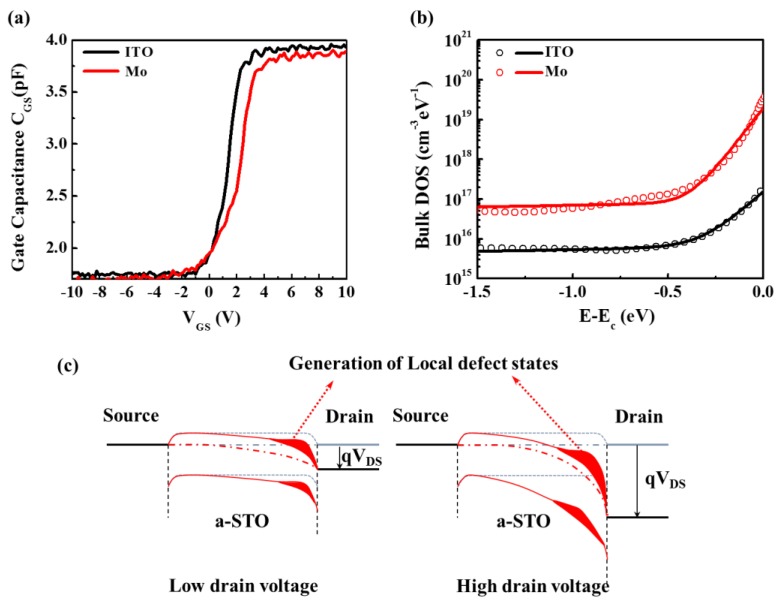
(**a**) Gate capacitance versus gate voltage (*C*_GS_-*V*_GS_) characteristics of a-STO TFTs with different electrodes at 10k Hz. (**b**) Extracted (symbols) and fitted by (5) (solid lines) DOS as a function of *E*-*E*_C_. (**c**) The schematic diagram of local defect states generation.

Nevertheless, it was a macro-explanation for the difference of the electrical performances of devices with different S/D electrode contacts. As known to all, the intrinsic properties of AOS-TFTs are attributed to subgap DOS. Therefore, it is meaningful to investigate the DOS of a-STO TFTs to further understand the influence of the electrodes on the device performance. The DOS of a device with different electrodes are extracted by using an analytical and simple method avoiding various complex extraction methods, such as numerical iterative solutions, temperature-dependent characteristics, or multi-frequency C–V characteristics. This simple method considers the low-frequency C–V characteristics of TFTs, which can be obtained by a small AC signal superimposed to the DC bias voltage in the condition of source and drain electrodes connected together [16]. Figure 4a shows the gate capacitance versus the gate voltage (*C*_GS_-*V*_GS_) characteristics of same dimension a-STO TFTs with ITO and Mo electrodes. The flat band voltage *V*_fb_ can be extracted by the I–V and C–V characteristics, following the method developed by Migliorato et al. [17] Figure 4b shows the DOS with respect to (*E*-*E*_C_) plot for a device with different electrodes contact (*E* is the state energy and *E*_C_ is the conduction band minimum). It was found that the distribution for the trap densities of states far from the *E*_C_ was quite flat in the energy gap for such metal oxide TFTs, but became larger near the *E*_C_. The DOS of a-STO TFTs could be divided into two parts: deep states and tail states. The plots were approximately fitted using the following superposition of the exponential tail states and exponential deep states:

(5) $$N_{t}\left(E\right)=N_{\mathrm{DA}}\exp\left(\frac{E-E_{C}}{E_{\mathrm{DA}}}\right)+N_{\mathrm{TA}}\exp\left(\frac{E-E_{C}}{E_{\mathrm{TA}}}\right)$$

where *N*_DA_/*N*_TA_ is the density of deep/tail states at the conduction edge, and *E*_DA_/*E*_TA_ is the characteristics energy of deep/tail states. The *N*_DA_/*N*_TA_ is extracted by extrapolating the deep/tail states to *E* = *E*_C_, while *E*_DA_/*E*_TA_ is extracted from the slope of log(*N*_t_) versus (*E*-*E*_C_) for the deep/tail states. In the AOS, the tail states are caused by structural disorder, which is characterized by a sharp conduction band tail slope because of the large overlapping s orbitals of the heavy cation. The deep states can be originated in excess oxygen in the thin film which forms deep acceptor states [18], because excess oxygen in the thin film can capture the electrons through $O^{0}+e^{-}\to O^{1-}$ and/or $O^{1-}+e^{-}\to O^{2-}$. The extracted values of the DOS with different electrodes are list in Table 2. In consideration of the near consistency of the results in Figure 3, the DOS of both devices could be speculated to be the same. However, it was clearly found that the deep/tail states’ densities of the a-STO TFTs with ITO contact were 6.5 × 10^15^/1.7 × 10^17^ cm^−3^ eV^−1^, which was significantly lower than those with Mo contact (8.5 × 10^16^/9.5 × 10^18^ cm^−3^ eV^−1^). Considering the almost identical *SS* values of the two devices, the different DOS was caused by the generation of local defect states near the electrode. The local defect states might be originated by two factors: (1) the formation of dangling bonds at the electrode–a-STO interface; in fact, on the basis the BDE of Mo-O (597 kJ/mol), Sn-O (528 kJ/mol), and In-O (346 kJ/mol), the electrode could bond with oxygen from a-STO during the high-temperature annealing process, which would result in dangling bonds that can cause various defect states [19]. A slight difference in the *μ*_FE_ of the two devices was primarily attributed to the existence of dangling bonds at the electrode/a-STO interface; (2) the generation of hot electrons during the operation of the device. The acceptor-like defect states were formed by hot electrons in the channel when a high *V*_DS_ was applied, which could capture the electrons. There was a distinction in the *μ*_sat_ of two devices, which was mainly ascribed to the generation of different magnitudes of acceptor-like defect states that could hinder the flow of electrons [20,21].

**Figure 5:**
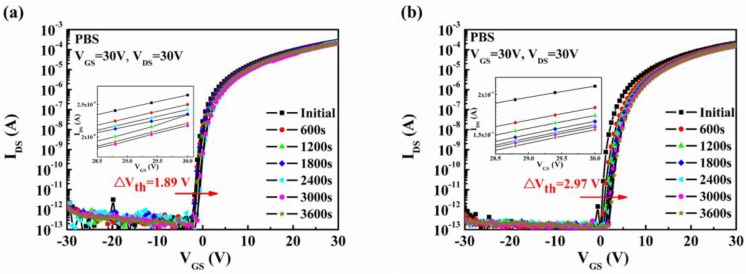
The evolution of time-dependent transfer curves under positive gate bias stress for a-STO TFTs with different S/D electrodes: (**a**) ITO, (**b**) Mo. The inserts show the variation of the on-current as a function of time.

At last, the stabilities of a-STO TFTs with ITO and Mo were investigated by positive gate bias stress (PBS), as shown in Figure 5. The devices were stressed under the condition *V*_GS_ = *V*_DS_ = 30 V, which was applied for 3600 s. In the case of PBS stability, the TFT with ITO contact (∆*V*_th_ = 1.89 V) exhibited a more stable behavior than that with Mo contact (∆*V*_th_ = 2.97 V). The reason for the positive shifts of *V*_th_ is generally the trapping of electrons at the gate insulator–semiconductor interface. Interestingly, the on-current of both devices decreased as the time increased. This, again, implied that the generation of the local defect states near the electrode, resulting in the formation of a potential barrier, could block the injection of electrons from the electrode.

## 4. Conclusions

The contact characteristics of a-STO TFTs employing ITO and Mo electrodes were investigated. Through TLM analysis, the formation of Ohmic contact and a good electrical performance were obtained in the device with ITO or Mo contact. Further, the DOS of the acceptor-like states of a-STO TFTs with ITO and Mo were extracted. The deep/tail states’ densities of the a-STO TFTs with ITO (6.5 × 10^15^/1.7 × 10^17^ cm^−3^eV^−1^) was lower than those with Mo, which was attributed to the generation of local defect states near the electrode. The local defect states could induce the formation of a potential barrier blocking the injection of electrons from the electrode.

## Figures and Tables

**Table 1:** Comparative table of device performance for amorphous STO-TFTs with ITO and Mo electrodes.

S/D Electrode	*μ*_FE_ (cm^2^/V s)	*μ*_sat_ (cm^2^/V s)	*V*_on_ (V)	*I*_on_/*I*_off_	*SS* (V/Decade)
ITO	5.6 ± 0.9	9.8 ± 0.6	−2.4 ± 0.5	(1.5 ± 0.6) × 10^9^	0.3 ± 0.1
Mo	5.4 ± 0.2	7.0 ± 0.7	0.2 ± 0.3	(8.1 ± 0.5) × 10^8^	0.3 ± 0.1

*μ*_FE_: field-effect mobility, *μ*_sat_: saturation mobility, *V*_on_: turn-on voltage, *I*_on_/*I*_off_: on/off current ratio, *SS*: subthreshold swing.

**Table 2:** The DOS parameters fitted by Equation (5) for different electrodes.

S/D Electrode	*N*_DA_ (cm^−3^eV^−1^)	*E*_DA_ (eV)	*N*_TA_ (cm^−3^eV^−1^)	*E*_TA_ (eV)
ITO	6.5 × 10^15^	5.0	1.7 × 10^17^	0.1
Mo	8.5 × 10^16^	4.2	9.5 × 10^18^	0.09

S/D Electrode: Source/Drain electrode, *N*_DA_: the density of deep states at the conduction edge, *E*_DA_: the characteristics energy of deep states, *N*_TA_: the density of tail states at the conduction edge, *E*_TA_: the characteristics energy of tail states.

## Supplementary Materials

The following are available online at http://www.mdpi.com/2079-4991/8/5/293/s1, Figure S1: (a) The work function of a-STO film (200 nm) measured by X-ray Photoelectron Spectroscopy. (b) Theoretically, the energy band diagram of a-STO film contacted with different electrodes: ITO and Mo. The work functions of ITO, Mo, and a-STO are 4.5 eV, 4.6 eV and 5.38 eV, respectively. Figure S2: I–V curves of a-STO film in contact with different electrodes: (a) ITO and (b) Mo. The linear I–V curve of TFT with ITO or Mo contact indicated that Ohmic contact was formed at the electrode–a-STO interface. Figure S3: The thickness of a-STO film tested by X-ray reflectivity (XRR). Figure S4: (a) The schematic diagram of a-STO TFT. (b) Cross-sectional high-resolution transmission electron microscope (HRTEM) image and elements distribution detected by energy-dispersive X-ray spectroscopy (EDS) mapping scan for a-STO TFT. Figure S5: (a) Cross-sectional transmission electron microscope (TEM) image and (b) elements distribution detected by energy-dispersive X-ray spectroscopy (EDS) line scan for a-STO TFT. An explanation of the subgap density of states (DOS) as an important parameter in AOS devices. The procedure for the extraction of the parameters from Equations (1) and (2) is described. The procedure for the proposed extraction method is described. Table S1: The properties (density, thickness, and roughness) of a-STO films.
