# Supplementary material for: Effect of Source/Drain Electrodes on the Electrical Properties of Silicon–Tin Oxide Thin-Film Transistors
Source: Nanomaterials (Basel). 2018 May 2;8(5):293. doi: 10.3390/nano8050293 (PMC5977307; doi:10.3390/nano8050293)
Supplement: Supplementary file 1 [file nanomaterials-08-00293-s001.pdf]

Effect of Source/Drain Electrodes on Electrical Properties of Silicon-Tin-Oxide Thin-Film Transistors

Xianzhe Liu[1], Honglong Ning[1], Weifeng Chen[1], Zhiqiang Fang[2], Rihui Yao[1,*], Xiaofeng Wang[3], Yuxi Deng[1], Weijian Yuan[1], Weijing Wu[1,*], Junbiao Peng[1]

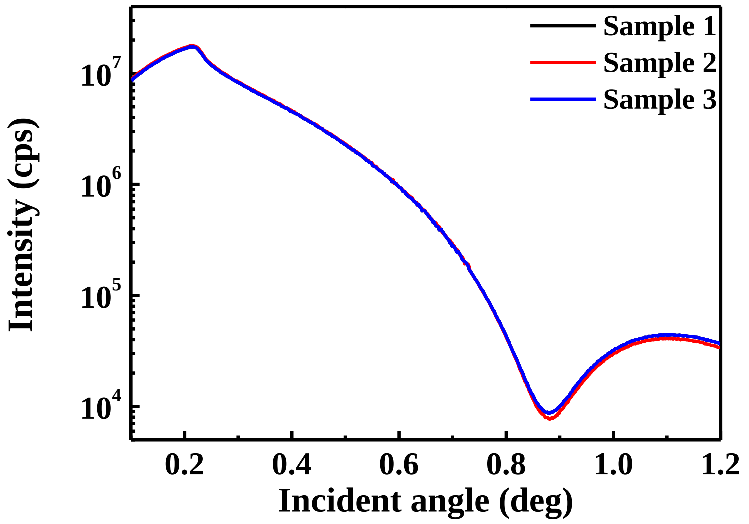

Figure S1. X-ray reflectivity (XRR) curves for a-STO films.

Table S1. The properties (density, thickness and roughness) of a-STO films.

Sample	Density (g/cm^3)	Thickness (nm)	Roughness (nm)
1	6.21	5.062	0.669
2	6.198	5.06	0.623
3	6.138	5.043	0.671

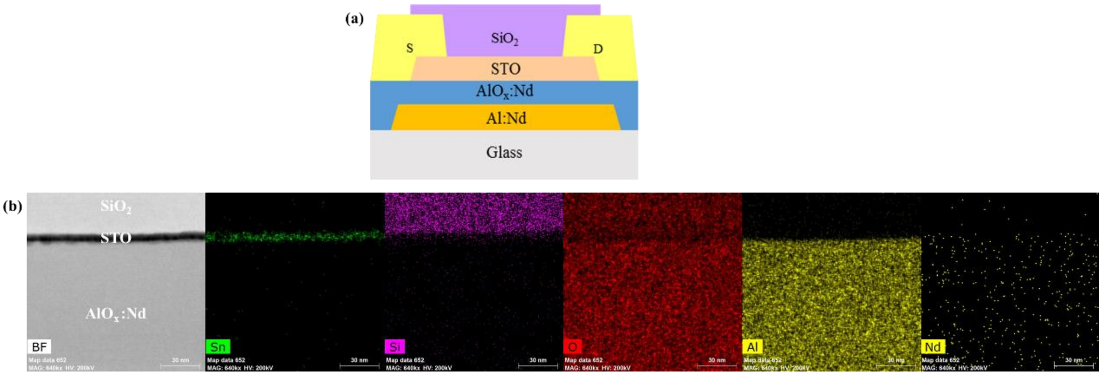

Figure S2. (a) The schematic diagram of a-STO TFT. (b) Cross-sectional high resolution transmission electron microscope (HRTEM) image and elements distribution detected by Energy-dispersive X-ray spectroscopy (EDS) mapping scan for a-STO TFT.

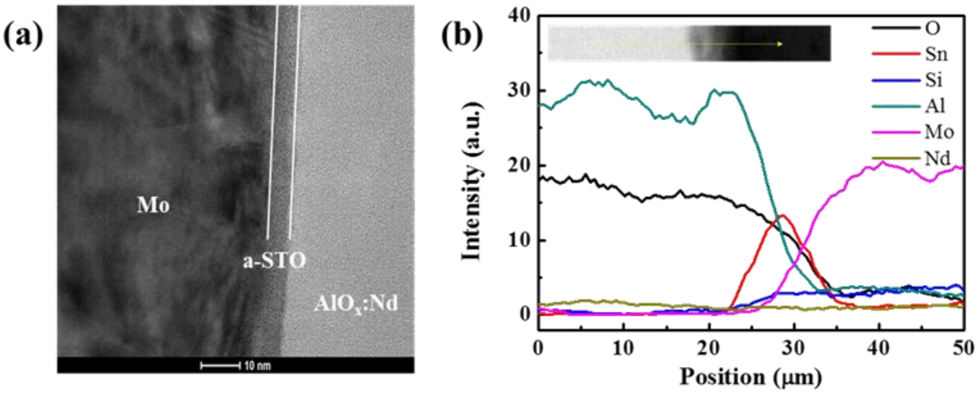

Figure S3. (a) The cross-sectional transmission electron microscope (TEM) image and (b) elements distribution detected by Energy-dispersive X-ray spectroscopy (EDS) line scan for a-STO TFT.

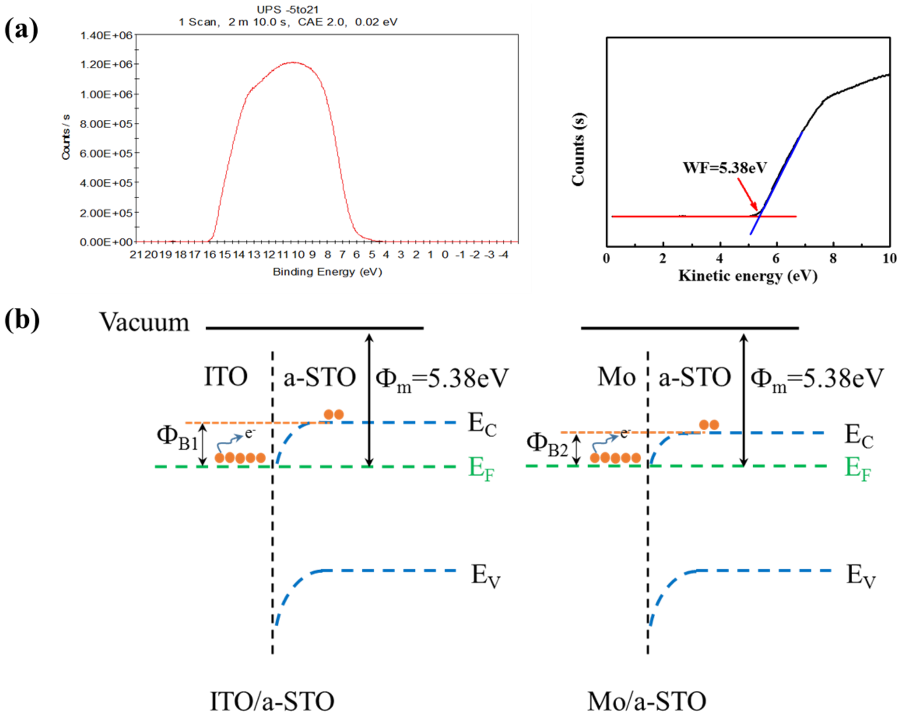

Figure S4. (a) The work function of a-STO film (200nm) measured by X-ray Photoelectron Spectroscopy. (b) Theoretically, the energy band diagram of a-STO film contacted with different electrodes: ITO and Mo. The work functions of ITO, Mo and a-STO are 4.5 eV, 4.6 eV and 5.38 eV, respectively.

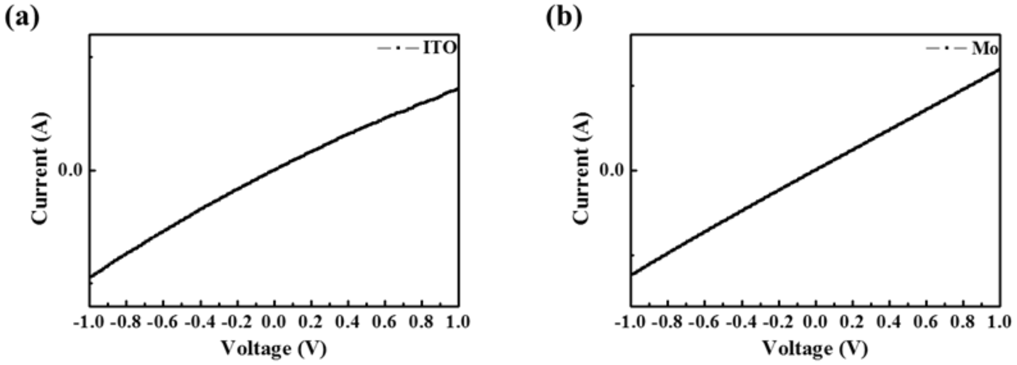

Figure S5. I-V curves of a-STO film contacted with different electrodes: (a) ITO and (b) Mo. The linear I-V curve of TFT with ITO or Mo contact indicated that Ohmic contact was formed at the electrode/a-STO interface.

The importance of DOS in AOS device:

In AOS devices, the subgap density of states (DOS) is an important parameter which plays a major part in controlling the mobility, the operation voltage and subthreshold swing of TFTs. The TFT mobilities are deteriorated by the subgap density of states (DOS), as roughly expressed by

$$\mu_{FE} = \mu \frac{N_{GS} - N_T}{N_{GS}}$$

where N_T is the total DOS of the unoccupied subgap DOS, and $N_{GS} = Cg(V_{GS} – V_{th})$ is the electron density induced by V_{GS}. The subgap DOS also determines the operation voltage of the TFT; the operation voltage is limited by the V_{GS} range required to switch the TFT from the off state to the on state, which is expressed by the S value defined by

$$\mathrm{S} = \frac{dV_{GS}}{dlogI_{DS}} = ln10\frac{k_B T}{e}(1 + \frac{eD_{sg}}{C_g})$$

D_{sg} is the subgap DOS at the Fermi level (E_F). These results indicate that reduction of the subgap DOS is the most important issue for realizing high-performance TFTs.

The extracted procedure of parameters from Equation 1 and Equation 2:

Equation 1:

$$I_{DS} = \frac{W}{L}\mu_{FE}C_i\left(V_{GS} - V_{th} - \frac{1}{2}V_{DS}\right)V_{DS}$$

$$I_{DS} = \frac{W}{L}\mu_{FE}C_i\left[(V_{GS} - V_{th})V_{DS} - \frac{1}{2}V_{DS}^2\right]$$

The $\frac{1}{2}V_{DS}^2$ can be ignored in consideration of the very small value of V_{DS} (~0.1V).

$$I_{DS} = \frac{W}{L}\mu_{FE}C_i(V_{GS} - V_{th})V_{DS}$$

V_{th} can be determined by using linear extrapolation of the tested transfer curve (I_{DS}-V_{GS}). The slope can also be obtained from linear extrapolation.

$$\mathrm{Slope} = \mu_{FE}\frac{W}{L}C_iV_{DS}$$

$$\mu_{FE} = \frac{L \cdot \text{Slope}}{W C_i V_{DS}}$$

Equation 2:

$$I_{DS} = \frac{W \mu_{sat} C_i}{2L} (V_{GS} - V_{th})^2$$

$$\sqrt{I_{DS}} = \sqrt{\frac{W \mu_{sat} C_i}{2L}} (V_{GS} - V_{th})$$

V_{th} can be determined by using linear extrapolation of the tested transfer curve ($\sqrt{I_{DS}}$-V_{GS}). The slope can also be obtained from linear extrapolation.

$$\text{Slope} = \sqrt{\frac{W \mu_{sat} C_i}{2L}}$$

$$\mu_{sat} = \frac{2L \cdot \text{Slope}^2}{W C_i}$$

The procedure for the proposed extraction method is described as follows.

$$\psi_s = \int_{V_{fb}}^{V_{gs}} \left(1 - \frac{C_g(V'_{gs})}{C_{ox}}\right) dV'_{gs} \qquad (1)$$

$$\rho(\psi_s) = -\frac{C_g(V_{gs}) \int_{V_{fb}}^{V_{gs}} C_g(V'_{gs}) dV'_{gs}}{\varepsilon_s \left(1 - \frac{C_g(V_{gs})}{C_{ox}}\right)} \qquad (2)$$

$$N_t(E_{F0} + q\psi_s) = -\frac{1}{q^2} \frac{\rho(\psi_s + \Delta\psi_s) - \rho(\psi_s)}{\Delta\psi_s} - \frac{n_0}{qV_t} exp\left(\frac{\psi_s}{V_t}\right) \quad (3)$$

Firstly, Ψ_s in terms of V_{gs} is calculated from the C_g-V_{gs} characteristics of TFTs by (1). Secondly, the surface charge concentration $\rho(\Psi_s)$ can be obtained from the C_g-V_{gs} characteristics of TFTs by (2). Finally, the density of states $N_t(E)$ with respect to some energy level ($E = E_{F0} + q\Psi_s$) can be extracted by (3). As seen above, the proposed extraction method of DOS has the advantages of analyticity and simplicity.
